# Training-Specific Changes in Regional Spontaneous Neural Activity Among Professional Chinese Chess Players

**DOI:** 10.3389/fnins.2022.877103

**Published:** 2022-05-27

**Authors:** Dongmei Liang, Lihua Qiu, Xujun Duan, Huafu Chen, Chengyi Liu, Qiyong Gong

**Affiliations:** ^1^School of Physical Education and Sports Exercise, South China Normal University, Guangzhou, China; ^2^National Demonstration Center for Experimental Sports Science Education, South China Normal University, Guangzhou, China; ^3^Department of Radiology, The Second People’s Hospital of Yibin, Yibin, China; ^4^Department of Radiology, Huaxi MR Research Center (HMRRC), West China Hospital of Sichuan University, Chengdu, China; ^5^Key Laboratory for Neuroinformation of Ministry of Education, School of Life Sciences and Technology, University of Electronic Science and Technology of China, Chengdu, China; ^6^Research Unit of Psychoradiology, Chinese Academy of Medical Sciences, Chengdu, China

**Keywords:** board games, Chinese chess, regional homogeneity, automaticity, Chinese language cognition, AD prevention

## Abstract

**Background:**

Our previous reports reflected some aspects of neuroplastic changes from long-term Chinese chess training but were mainly based on large-scale intrinsic connectivity. In contrast to functional connectivity among remote brain areas, synchronization of local intrinsic activity demonstrates functional connectivity among regional areas. Until now, local connectivity changes in professional Chinese chess players (PCCPs) have been reported only at specific hubs; whole-brain-based local connectivity and its relation to training profiles has not been revealed.

**Objectives:**

To investigate whole-brain local connectivity changes and their relation to training profiles in PCCPs.

**Methods:**

Regional homogeneity (ReHo) analysis of rs-fMRI data from 22 PCCPs versus 21 novices was performed to determine local connectivity changes and their relation to training profiles.

**Results:**

Compared to novices, PCCPs showed increased regional spontaneous activity in the posterior lobe of the left cerebellum, the left temporal pole, the right amygdala, and the brainstem but decreased ReHo in the right precentral gyrus. From a whole-brain perspective, local activity in areas such as the posterior lobe of the right cerebellum and the caudate correlated with training profiles.

**Conclusion:**

Regional homogeneity changes in PCCPs were consistent with the classical view of automaticity in motor control and learning. Related areas in the pattern indicated an enhanced capacity for emotion regulation, supporting cool and focused attention during gameplay. The possible participation of the basal ganglia-cerebellar-cerebral networks, as suggested by these correlation results, expands our present knowledge of the neural substrates of professional chess players. Meanwhile, ReHo change occurred in an area responsible for the pronunciation and reading of Chinese characters. Additionally, professional Chinese chess training was associated with change in a region that is affected by Alzheimer’s disease (AD).

## Introduction

Chess serves studies in cognitive science as *Drosophila* serves studies in biological science. Many processes, such as perception (Sheridan and Reingold, 2017), memory (Gong et al., 2015), problem solving (Pereira et al., 2020), and empathy (Powell et al., 2017), become more apparent in the classical research paradigm of chess. With the application of non-invasive imaging techniques (Nichelli et al., 1994; Onofrj et al., 1995; Amidzic et al., 2001; Silva et al., 2018; Fuentes-García et al., 2019; Pereira et al., 2020) and especially functional magnetic resonance imaging (fMRI) (Atherton et al., 2003; Bilalić et al., 2011; Wan et al., 2011; Duan et al., 2014; Hänggi et al., 2014; Premi et al., 2020; Wang et al., 2020) in the human brain, neural substrates of cognitive processes have gradually been revealed. In studies investigating these substrates, cognitive research on expertise was a main research domain. Based on the theory of chunks in chess experts (Chase and Simon, 1973), the medial temporal lobe was initially revealed as the basis of long-term memory (LTM) in chess experts (Amidzic et al., 2001), and then the caudate region was comprehensively shown to be responsible for automatically producing the best next move in board games (Wan et al., 2011). Specific regions have long been believed to form the basis of cognitive expertise in board games. However, from the investigation of functional connectivity between the caudate and the default mode network (DMN) (Duan et al., 2012a), step-by-step exploration of specific region-based functional connections was applied (Duan et al., 2014; Sohn et al., 2017; Song et al., 2020; Wang et al., 2020). Recently, whole-brain-based detection of brain functional connectivity was reported as a dynamic functional network characteristic of Chinese chess experts (Premi et al., 2020).

Since the 1st China National Mind Sports Games in 2010 in Chengdu, China, the brain characteristics associated with cognitive expertise in Chinese chess have been discussed throughout the scientific world (e.g., Duan et al., 2012a,b, 2014; Premi et al., 2020; Song et al., 2020; Wang et al., 2020). Chinese chess (*Xiangqi* in Chinese) is a traditional board game originating from military strategies in ancient China. To the best of our knowledge, this game was first introduced in an English publication in 1895 (Platt, 1895). As in chess, the records of professional players in each competition are compiled to assign ranking points to each player, reflecting the person’s skill level. Xiangqi is remarkable among board games in that its famous endgame problems [such as “wild horses run on the farm” (Hung et al., 2017)], moving rules (such as “the horse moves in the shape of the character “RI” and “the elephant moves in the shape of the character “TIAN”)^1^ and combat strategies (such as “dāng tóu pào, m ǎ lái tiào”; See text footnote) are described and taught in vivid sentences (“rhymes” or “sayings”) or descriptive battle stories, which may explain the special relationship of this game to the cognitive processes of Chinese language cognition, sematic memory (SM) and episodic memory (EM). Changes in SM and EM are both early markers of Alzheimer’s disease (AD) (Marra et al., 2016; Gagliardi et al., 2019; Venneri et al., 2019). Recently, some reports have discussed the AD-preventive effects of board games (Nakao, 2019; Qureshi, 2019). For example, a previous randomized clinical trial of 147 AD patients reported that AD symptoms were reduced in patients who played the game of Go (Lin et al., 2015).

Our previous studies (Duan et al., 2012a,b, 2014) reported morphological changes in the caudate, the enhanced connectivity of the caudate to the DMN, and remote functional connectivity alterations including different global topological properties of the whole-brain functional networks and intrinsic brain networks. These reports reflected some aspects of neuroplastic changes from long-term Chinese chess training but were mainly based on large-scale intrinsic connectivity. In contrast to functional connectivity among remote brain areas, synchronization of local intrinsic activity demonstrates functional connectivity among regional areas (Jia et al., 2017). Until now, local connectivity changes in professional Chinese chess players (PCCPs) have been reported only at specific hubs (Song et al., 2020); whole-brain-based local connectivity and its relation to training profiles has not been revealed.

Regional homogeneity (ReHo), revealing the homogeneous characteristics of local brain activity, is one kind of postprocessing method of local spontaneous activity. ReHo is based on Kendall’s coefficient concordance (KCC) to measure the similarity of the time series of a given voxel to those of its nearest neighbors in a voxel-wise way in rs-fMRI analysis (Zang et al., 2004). Recently, some reports (e.g., Jiang et al., 2015; Jiang and Zuo, 2016) revealed the neurobiological relevance underlying ReHo, including anatomical morphology, brain development, and neurocognitive factors. Therefore, ReHo was confirmed to be a useful neuroimaging tool to understand human brain function (He et al., 2007; Wang et al., 2011; Wu et al., 2011; Dai et al., 2012; Tian et al., 2012; Dong et al., 2014; Liu et al., 2015; Lv et al., 2019). As one morphological change typical of PCCPs was detected in the caudate (Duan et al., 2012a), which subserves the associative phase of cognitive procedural learning (Chiu et al., 2017), we might expect ReHo changes to be mostly similar to their structural and functional equivalents in neuroimaging studies that have revealed training-specific areas.

In this study, ReHo analysis was performed based on rs-fMRI data to explore the whole-brain local functional connectivity changes in PCCPs and the relation of these changes to training profiles.

## Materials and Methods

### Participants

A total of 43 subjects were included in the present study. One group included 22 PCCPs (14 males and 8 females; age, 27.32 ± 8.31 years; years of education, 13.45 ± 2.37; rating points, 2410 ± 116; professional training years, 10 ± 9.32; and professional training hours per day, 4.25 ± 1.82). Another group included 21 novices (13 males and 8 females; age, 26.20 ± 8.17 years; years of education, 13.38 ± 3.37) who knew the rules of the game and simple strategies but with no game experience (Campitelli et al., 2005). PCCPs and novices were sex-, education- and age-matched. To further examine the difference between PCCPs and novices, both groups were tested by Raven’s Standard Progressive Matrices, and two groups did not differ on general intelligence (*P* = 0.63, two tailed *t-*test). All participants had normal or corrected-to-normal vision. Written informed consent was obtained from all subjects. The proposal was approved by the local Ethics Committee of Huaxi Hospital, Sichuan University.

### Data Acquisition

Images were acquired using a 3.0T Siemens Magnetom Trio scanner in the Huaxi MR Research Center. Functional images were acquired using a single-shot, gradient-recalled echo-planar imaging sequence [repetition time (TR) = 2,000 ms, echo time (TE) = 30 ms and flip angle = 90^^°^]. Thirty transverse slices [field of view (FOV) = 24 cm, in-plane matrix = 64 × 64, slice thickness = 5 mm, without gap, voxel size = 3.8 × 3.8 × 5] and 205 volumes were obtained from each subject. The first five volumes were discarded to ensure steady-state longitudinal magnetization. During the scanning procedure, a standard head coil with foam padding was used to restrict head motion. Subjects were instructed simply to rest with their eyes closed, not to think of anything in particular and not to fall asleep.

### Data Analysis

Image pre-processing was performed using SPM8 software.^2^ The first five volumes were discarded to ensure steady-state longitudinal magnetization. The remaining 200 volumes were first corrected for the temporal difference and head motion. In this study, the threshold for head motion was lower than ±1.5 mm or ±1.5^^°^. We calculated frame-wise displacement (FD) which reflected the head movement at every different time point by employing 6 displacements from the rigid body motion correction procedure (Power et al., 2012), and found no significant differences (*P* = 0.82) in FD between PCCPs (0.159 ± 0.16 mm) and novices (0.150 ± 0.07 mm) using two-sample *t*-tests. The resulting images were then normalized to the standard SPM8 echo-planar imaging template and resampled to a standard stereotaxic space at a resolution of 3 mm × 3 mm × 3 mm. Finally, the normalized images were temporally band-pass filtered (0.01 < f < 0.08 Hz) to reduce the effects of low-frequency drift and high-frequency physiology noise (Biswal et al., 1995); additionally, the linear trend was removed.

### Regional Homogeneity Analysis

The KCC (Kendall and Gibbons, 1990) was calculated to measure the similarity of the time series of a defined cluster. In the present study, 27 nearest neighbor voxels were defined as a cluster. The KCC was given to the center voxel (Zang et al., 2004) as follows:


$$W=\frac{\sum {\left(R_{i}\right)}^{2}-n\,{\left(\bar{R}\right)}^{2}}{\frac{1}{12}\,K^{2}\,\left(n^{3}-n\right)}$$

where *W* is the KCC among given voxels, ranging from 0 to 1; *R*_*i*_ is the sum rank of the *i*^*th*^ time point; $\bar{R}=\left(n+1\right)\,K/2$ is the mean of the *R*_*i*_ values; *K* is the number of time series points within a measured cluster; and *n* is the number of ranks (here, *n* = 200 time points). REST software (Resting-state fMRI data analysis toolkit)^3^ was used to calculate individual ReHo values in a voxel-wise way. Each individual ReHo map was divided by that subject’s global mean KCC value within the brain mask. The ReHo maps were then spatially smoothed with a Gaussian filter of 8 mm of full width at half maximum (FWHM).

### Second-Level Analysis

Group statistical analysis was performed in SPM8. The one-sample *t*-tests results from the two groups were combined to get a new map. By binarizing the map, a combined explicit mask was obtained. The significance threshold was set at *P* < 0.01, corrected by the false discovery rate (FDR) criterion (Genovese et al., 2002).

Then, two-sample *t*-tests were performed to show the between-group difference in ReHo. The *t*-map was created with a combined threshold of *P* < 0.005 and a minimum cluster size of 46 voxels using the AlphaSim program in REST software. This approach applied Monte Carlo simulation to calculate the probability of false positive detection by taking into consideration both the individual voxel probability threshold and cluster size.

Additionally, to explore whether ReHo correlates with the rating points, years of professional training and training intensity of PCCPs, a correlation analysis of ReHo versus these training profiles was performed in this group for each voxel of the whole brain. For the correlation analyses, we set the significance threshold at *P* < 0.05 (combined threshold of *P* < 0.001 and a minimum cluster size of 20 voxels, corrected by AlphaSim).

## Results

### Within-Group Results

**FIGURE 1:**
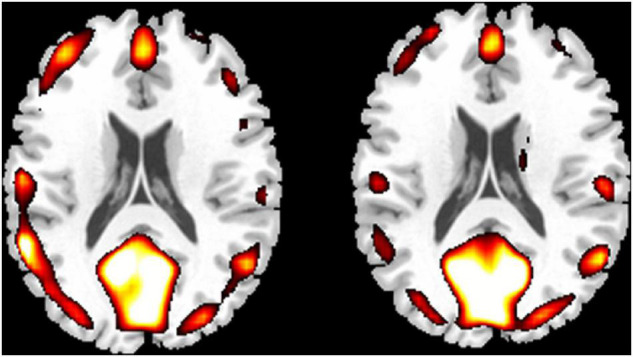
One-sample results for novices (left column) and professional Chinese chess players (PCCPs) (right column). The regions indicated by a warm color showed greater regional homogeneity (ReHo). The threshold was *P* < 0.01, FDR corrected. The left side of the image corresponds to the left side of the brain.

In order to intuitively display ReHo results for Chinese chess novices and PCCPs, a ReHo map was calculated within each group and shown in Figure 1 (one-sample *t*-test; *P* < 0.01, corrected by FDR). For visual observation, areas in the DMN (Raichle et al., 2001) including the posterior cingulate cortex/precuneus (PCC/Pcu), medial prefrontal cortex (MPFC) and bilateral inferior parietal lobe (IPL) displayed significantly greater ReHo than other regions.

### Between-Group Results

**FIGURE 2:**
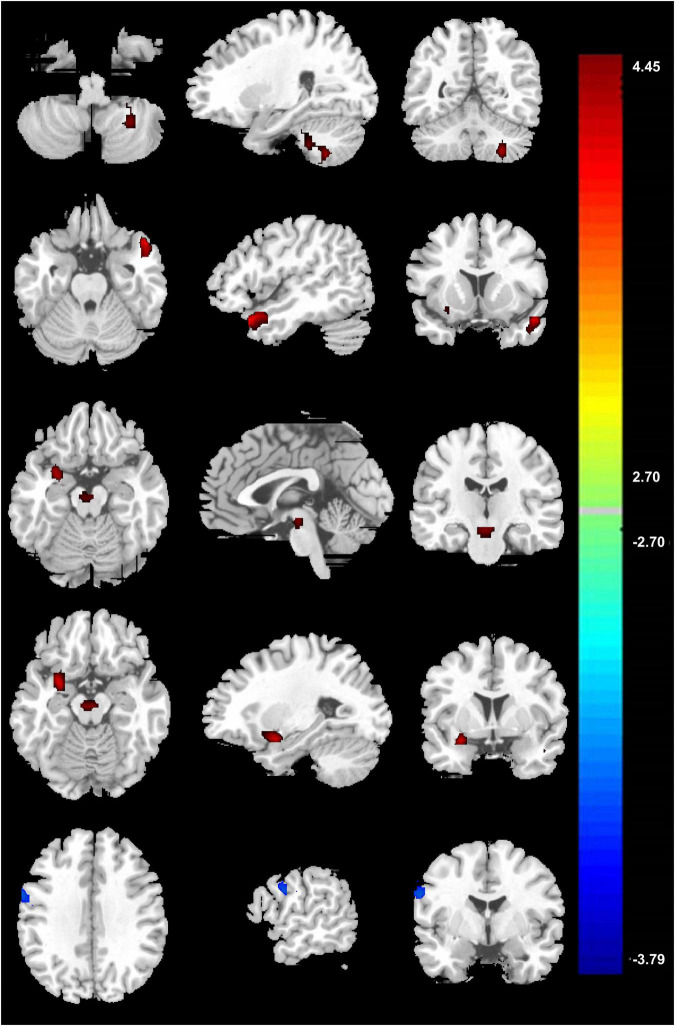
Two-sample result between PCCPs and novices (*P* < 0.05, AlphaSim corrected, a combined threshold of *P* < 0.005, and a minimum cluster size of 46 voxels). Hot and cold colors indicate ReHo increases and decreases in PCCPs, respectively, and the number indicates the *t-*value. The left and right sides in this figure correspond to the right and left sides of the brain, respectively. Further details of these regions are presented in Table 1.

Compared with the novices, PCCPs revealed increased ReHo in the left cerebellum posterior lobe, left temporal pole, right amygdala, and brainstem and decreased ReHo in the right precentral gyrus (two-sample *t*-test, *P* < 0.05, corrected by AlphaSim; Figure 2 and Table 1).

**TABLE 1:** Comparison of regions with increased/decreased regional homogeneity (ReHo) in professional Chinese chess players (PCCPs) compared to novices.

Anatomical region	MNI (*x*,*y*,*z*)^*a*^	BA	Voxels	*t* ^*b*^
**Increased ReHo regions**				
L cerebellum posterior lobe	−27, −57, −51	–	125	3.94
L temporal pole	−48, 18, −27	38	104	4.45
R amygdala	27, 0, −18	–	79	4.04
brainstem	3, −21, −18		60	3.50
**Decreased ReHo regions**				
R precentral gyrus	63, 3, 30	6	90	−3.68

*MNI, Montreal Neurologic Institute; BA, Brodmann’s area; L, left; R, right.*

*ReHo, regional homogeneity; MNI, Montreal Neurologic Institute; BA, Brodmann’s area; L, left; R, right.*

*^a^Coordinates of primary peak locations in MNI space; ^b^Represents the statistical value of the peak voxel showing ReHo differences between groups. In the PCCP group, a positive t-value represents increased ReHo, and a negative t-value represents decreased ReHo.*

### Correlational Results

**FIGURE 3:**
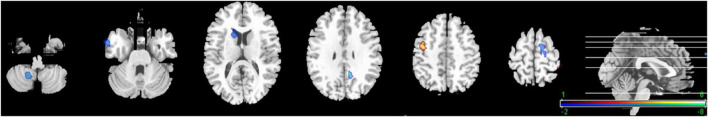
Significant correlations between ReHo and training profiles in PCCPs (*P* < 0.05, AlphaSim corrected, a combined threshold of *P* < 0.001, and a minimum cluster size of 20 voxels). Hot and cold colors indicate positive and negative correlations, respectively. The numbers on the right color-bar refer to the *t-*value. The left and right sides in this figure correspond to the right and left sides of the brain, respectively. Further details of these regions are presented in Table 2.

In the voxel-based group comparisons of ReHo maps, we chose a statistical threshold at voxel level *P* < 0.05 (AlphaSim corrected) with a minimum cluster size of 20 voxels to reduce Type I errors, resulting in a combined threshold of *P* < 0.001. The correlation of ReHo for each whole-brain voxel against rating points of PCCPs showed a significantly positive correlation in the right precentral gyrus and a significantly negative correlation in the left PCC/Pcu and right middle temporal gyrus (MTG). The professional training years of PCCPs were negatively correlated with the left SMA and right cerebellum posterior lobe. The ReHo of the right caudate was negatively correlated with training hours. Please see Figure 3 and Table 2 for more details.

**TABLE 2:** Significant correlations between ReHo and training profiles in PCCPs.

Anatomical region (BA areas)	MNI (*x*,*y*,*z*)^*a*^	Voxels	*t* ^*b*^
**Significant correlation between ReHo and rating points**			
R precentral gyrus (6)	39, −9, 45	35	5.11
L PCC/precuneus (31)	−12, −54, 27	40	−6.90
R middle temporal gyrus (21)	57, 0, −30	27	−6.58
**Significant correlation between ReHo and professional training years**			
L supplementary motor area (SMA) (6)	−15, −12, 63	26	−6.24
R cerebellum posterior lobe	15, −60, −42	79	−6.20
**Significant correlation between ReHo and professional training hours per day**			
R caudate	18, 12, 15	31	−4.00

*MNI, Montreal Neurologic Institute; BA, Brodmann’s area; L, left; R, right.*

*^a^Coordinates of primary peak locations in MNI space. ^b^Represents the peak statistical value of voxels showing ReHo correlated with training profiles. Positive and negative t-values indicate positive and negative correlations between ReHo and training profiles, respectively.*

## Discussion

Neuroscience studies have investigated the neural substrates of motor control and learning, which has shed light on the neural mechanism of expertise. From cognitive processes to associative processes and to automatic processes, changing patterns of skill performance are theoretically described; the main features of the automatic process are “*rapid, smooth, effortless, demand little intentional capacity and difficult to consciously disrupt”* (Yarrow et al., 2009). In the classical view of automaticity, it is believed that as long-term practice makes skills reflexive, subcortical structures primarily activate, whereas novel behaviors require attention and flexible thinking that depend on the cortex (Ashby et al., 2010). Pertinently, in reports on the brains of chess experts, this behavior was also described as follows: “*the pattern of activation moves from frontal parts at the beginning of the process to posterior parts responsible for retrieval of domain specific knowledge around the final expertise stage”* (Debarnot et al., 2014). In between-group analyses of this study, the ReHo of PCCPs decreased in the precentral gyrus but increased in the cerebellum, temporal pole, amygdala and brainstem; this pattern of activity was similar to the above-described changes.

A consensus has been reached on the relation between the cerebellum and emotion (Adamaszek et al., 2017). In addition to the cerebellum posterior lobe, we located areas of increased spontaneous brain activity in the temporal pole, amygdala and brainstem. The amygdala reflects emotion (Weymar and Schwabe, 2016), especially emotional regulation (Li et al., 2016; Morawetz et al., 2017) and cognitive reappraisal (d’Arbeloff et al., 2018). Notably, there is also a relation between the brainstem and emotion (Venkatraman et al., 2017). Also, the temporal pole is reported in the emotion of aggression (Breitschuh et al., 2018). As one dimension of personality, emotional expression control was once reported to incrementally contribute to the prediction of chess playing strength (Grabner et al., 2007). A randomized controlled trial protocol described a Go intervention programme designed to enhance elementary school students’ cognitive function and their capacity for emotional and behavioral control (Tachibana et al., 2012). In the current study, we deduce the possibility of enhanced emotion regulation function that results from long-term Chinese chess training, indicating superior emotion regulation ability that supports cool and focused attention in PCCPs during gameplay.

An ordinal consensus supports functional interactions between the basal ganglia and cortex and between the cerebellum and cortex. In the consensus view, the basal ganglia and the cerebellum are reported to form a densely interconnected network, namely, the basal ganglia-cerebellar-cerebral cortical networks, in which the caudate, different parts of the cerebellum and cortex form different networks supplying a neural basis for cognition (such as executive function) as well as for neuropsychiatric disorders (such as AD and anxiety) (Bostan and Strick, 2018). In addition to the negative correlation between the right caudate and training hours each day, we found a negative correlation between the right posterior lobe of the cerebellum and length of professional training and a positive correlation between the right precentral gyrus and rating points. All these findings demonstrate a similar pattern of concordant changes in some brain regions, such as the basal ganglia-cerebellar-cerebral networks in the right hemisphere, among PCCPs. These results add to our present knowledge of the neural substrates of professional chess players. Prior to this study, the caudate had been revealed initially as a neural substrate (Wan et al., 2011; Duan et al., 2012a,b), and the thalamus was recently reported as a neural substrate (as its mediation between the caudate and frontal cortex) (Wang et al., 2020). The present study reflects the participation of the basal ganglia-cerebellar-cerebral networks.

Previous studies reported that as motor skills became automatic, the activation of the SMA decreased (Poldrack et al., 2005; Puttemans et al., 2005). Reports from sequence training, task training and practice processes demonstrate a similar changing trend in the SMA (Nyberg et al., 2006; Hatanaka et al., 2009; Ma et al., 2010). Our result of a negative correlation between the SMA and training years is parallel to these conclusions. In addition to its role in training-specific plasticity, the left SMA plays an important role in phonological processing in Chinese language cognition (Kuo et al., 2004; Veroude et al., 2010); the left SMA also takes part in Chinese character reading (Tan et al., 2000; Kuo et al., 2003). Although the correlation between this training-specific area and language cognition is a novel finding in the study of the neural basis of board game experts, a previous report has already shown such a relationship in brain region mainly contributing to corresponding action to language understanding. For example, Beilock et al. (2008) reported that the left BA6 (dorsal lateral premotor cortex), as a region normally devoted to higher-level action selection and implementation, also supported specialized motor (sports) experience enhancing action-related language understanding even when there was no intention to perform a real action.

Chunking theory-induced neuroscience investigations have indicated that LTM chunks of domain-specific information are stored in the ventral areas of the temporal lobe, including the parahippocampal gyrus (PHG) and fusiform gyrus (Campitelli et al., 2007). The PHG plays an important role in EM (Di Paola et al., 2007; Gallagher and Koh, 2011); changes in EM serve as the earliest and “marker” cognitive function alterations in AD (Bäckman et al., 2001; Aretouli and Brandt, 2010; Gallagher and Koh, 2011; Marra et al., 2016; Gagliardi et al., 2019). Moreover, the connection of the PHG with the PCC/Pcu and MTG was positively correlated with the Mini-Mental State Examination (MMSE) score, indicating functional connectivity that reflects the progression of cognitive degeneration disease. Pertinently, the PHG is important in mediating the connectivity between the hippocampus and hubs of the DMN as well as the connection between the MTG memory system and the DMN (Liu et al., 2016). Both neuropathological (Van Hoesen et al., 2000) and structural MRI (Pantel et al., 2003) evidence have demonstrated that selective morphological brain changes and atrophy in the PHG represent a preclinical stage of AD. Recently, Jia et al. (2017) reported a link between local synchronization alterations in the PHG and APOE-related cerebral physiological heterogeneity. All related findings demonstrated the possibility that plastic ReHo changes in the PHG by professional Chinese chess training may indicate its role in AD prevention. A 5.1-year study of 469 elderly individuals, among whom dementia developed in 124 subjects (of which more than fifty percent developed AD), found that playing board games was associated with a reduced risk of dementia (Verghese et al., 2003). This protection was later explained by the cognitive reserve theory that the number and strength of neuronal connections in the brain could be increased by training; the more connections were built up, the larger reserves to counteract the rate at which neurons were disappearing from the brain as these neurons were destroyed by AD pathology (Marx, 2005). In this study, a negative correlation was found between ReHo of the right MTG and rating points, which demonstrates the relationship between the combined function of the PHG and fusiform gyrus and training-specific experiences among PCCPs.

## Conclusion

Through the comparison of ReHo analysis of rs-fMRI data between PCCPs and novices, training-specific whole-brain local connectivity changes in PCCPs were revealed. Brain ReHo of PCCPs demonstrated a similar changing pattern described in the classical view of automaticity in motor control and learning. Besides, some ReHo changes occurred in an area responsible for Chinese character pronouncing and reading. Moreover, professional Chinese chess training induced ReHo changes located in AD-related areas, which suggested the possibility of AD preventive effects from Chinese chess training. Findings from this study demonstrate the feasibility of using ReHo as a research tool to monitor board game-induced brain plastic changes and shed light on the neural substrates underlying cognition in Chinese chess playing.

## Data Availability Statement

Data are available in a public, open access repository at the International Neuroimaging Data-Sharing Initiative or INDI (http://fcon_1000.projects.nitrc.org/index.html).

## Ethics Statement

The studies involving human participants were reviewed and approved by the Local Ethics Committee of Huaxi Hospital, Sichuan University. The participants provided their written informed consent to participate in this study.

## Author Contributions

All authors listed have made a substantial, direct, and intellectual contribution to the work, and approved it for publication.

## Conflict of Interest

The authors declare that the research was conducted in the absence of any commercial or financial relationships that could be construed as a potential conflict of interest.

## Publisher’s Note

All claims expressed in this article are solely those of the authors and do not necessarily represent those of their affiliated organizations, or those of the publisher, the editors and the reviewers. Any product that may be evaluated in this article, or claim that may be made by its manufacturer, is not guaranteed or endorsed by the publisher.

## Footnotes

1
https://en.wikipedia.org/wiki/Xiangqi

2
http://www.fil.ion.ucl.ac.uk/spm

3
http://sourceforge.net/projects/resting-fmri
